# Entropy of eye movement during rapid automatized naming

**DOI:** 10.3389/fnhum.2022.945406

**Published:** 2022-08-04

**Authors:** Hongan Wang, Fulin Liu, Yuhong Dong, Dongchuan Yu

**Affiliations:** ^1^Key Laboratory of Child Development and Learning Science of Ministry of Education, School of Biological Science and Medical Engineering, Southeast University, Nanjing, China; ^2^Henan Provincial Medical Key Lab of Language Rehabilitation for Children, Sanmenxia Central Hospital, Sanmenxia, China; ^3^Henan Provincial Medical Key Lab of Child Developmental Behavior and Learning, The Third Affiliated Hospital of Zhengzhou University, Zhengzhou, China

**Keywords:** developmental dyslexia, rapid automatized naming (RAN), eye tracking, entropy, attention

## Abstract

Numerous studies have focused on the understanding of rapid automatized naming (RAN), which can be applied to predict reading abilities and developmental dyslexia in children. Eye tracking technique, characterizing the essential ocular activities, might have the feasibility to reveal the visual and cognitive features of RAN. However, traditional measures of eye movements ignore many dynamical details about the visual and cognitive processing of RAN, and are usually associated with the duration of time spent on some particular areas of interest, fixation counts, revisited fixation counts, saccadic velocities, or saccadic amplitudes. To cope with this drawback, we suggested an entropy-based method to measure eye movements for the first time, which first mapped eye movements during RAN in a time-series and then analyzed the time-series by a proper definition of entropy from the perspective of information theory. Our findings showed that the entropy was more sensitive to reflect small perturbation (e.g., rapid movements between focuses in the presence of skipping or omitting some stimulus during RAN) of eye movements, and thus gained better performance than traditional measures. We also verified that the entropy of eye movements significantly deceased with the age and the task complexity of RAN, and significantly correlated with traditional eye-movement measures [e.g., total time of naming (TTN)] and the RAN-related skills [e.g., selective attention (SA), cognitive speed, and visual-motor integration]. Our findings may bring some new insights into the understanding of both RAN and eye tracking technique itself.

## Introduction

Developmental dyslexia, involving genetic and environmental factors, is a hereditary and life-long neurodevelopmental disorder characterized by several deficits in reading and writing despite adequate intelligence (Association, 2013). Its etiology remains debated even after more than a century of research, but more and more evidences (Goswami, 2015; Ullman et al., 2020; Araújo et al., 2021) suggest that developmental dyslexia might be interpreted by: (i) Poor phonological awareness; or/and (ii) visual-attentional deficits and abnormal eye movement patterns. Both possibilities may explain that the individuals with developmental dyslexia usually have well-documented difficulties in visual naming, in addition to their difficulties in reading.

Rapid automatized naming (RAN) tasks (Wolf et al., 2000; Georgiou et al., 2008; Norton and Wolf, 2012; Snowling and Melby-Lervag, 2016; Araújo and Faísca, 2019; McWeeny et al., 2022) have been proposed for measuring individuals’ ability to retrieve and name a series of letters, numbers, objects, or colors sequentially as quickly as possible. RAN looks simple but is actually associated with a broad range of cognitive processes, including attention, executive functions (e.g., working memory, inhibitory control), and linguistic processes (e.g., phonological retrieval, visual-verbal connections) (Wolf et al., 2000; Georgiou et al., 2008; Norton and Wolf, 2012; Snowling and Melby-Lervag, 2016; Araújo and Faísca, 2019; McWeeny et al., 2022). Its clinical utility has been reported in the evaluation of several cognitive and neurobiological disorders, including developmental dyslexia (Goswami, 2015), specific language impairment (Snowling and Melby-Lervag, 2016), attention deficit/hyperactivity disorder (ADHD) (Tannock et al., 2000), and autism spectrum disorder (ASD) (Hogan-Brown et al., 2014; Zhao J. et al., 2019). Remarkably, more and more findings (Wolf et al., 2000; Georgiou et al., 2008; Norton and Wolf, 2012; Snowling and Melby-Lervag, 2016; Araújo and Faísca, 2019; McWeeny et al., 2022) have shown that the RAN deficits might become even more prominent in interpreting and characterizing the features of developmental dyslexia than other deficits in cognitive skills, such as phonological awareness, short-term memory, letter knowledge, and vocabulary. Furthermore, previous meta-analyses have documented the significant correlation between RAN and reading abilities across various reading constructs and languages (Swanson et al., 2003; Araújo et al., 2015; Hjetland et al., 2017). Hence, it is hypothesized that the visual and cognitive patterns or features during RAN might be promising predictors of reading abilities and developmental dyslexia in children (Swanson et al., 2003; Araújo et al., 2015; Hjetland et al., 2017).

Theoretically, to better understand the visual and cognitive patterns or features during RAN, one should monitor the focus points in sequence and record the essential ocular activities during RAN, such as duration of time spent on some particular areas of interest, rapid movements between focuses, scanning path, temporal-spatial series of focuses, and so on. However, traditional behavioral observation method cannot be competent for recording such the detailed temporal-spatial dynamical information during RAN (Wolf et al., 2000; Georgiou et al., 2008; Norton and Wolf, 2012; Snowling and Melby-Lervag, 2016; Araújo and Faísca, 2019; McWeeny et al., 2022). Fortunately, eye-tracking method (Armstrong and Olatunji, 2012; Chawarska et al., 2013; Lai et al., 2013), among others, has been intensively used in psychology for decades to reveal the fundamental cognitive processes and mechanisms involved in reading and visual perception, and thus has the feasibility to characterize the essential features of RAN. However, to date, there are only a few studies (Jones et al., 2008; Hogan-Brown et al., 2014; Araújo et al., 2021) that focus on analyzing eye movements during RAN.

The scales of eye-tracking measurements can be roughly divided into three categories: temporal, spatial and count (Weeks and Atlas, 2015). The temporal scales measure eye movements in a time dimension, e.g., duration of time spent on some particular areas of interest; the spatial scales measure eye movements in a space dimension, e.g., fixation position, fixation sequence, saccade length and scanpath patterns; and the count scales measure eye movements on a count or frequency basis, e.g., fixation counts and revisited fixation counts (Weeks and Atlas, 2015). However, thus far, traditional eye-movement measures (Wiig et al., 2000; Tran et al., 2014; Weeks and Atlas, 2015) have not considered fully the temporal-spatial patterns of eye movements from the perspective of non-linear dynamics or information theory. It is hypothesized that eye movements can be evaluated using more powerful strategies (e.g., non-linear time-series analysis).

On the other hand, the entropy, as a non-linear time-series analysis method, has been successfully applied to reflect the complexity and irregularity of a system from the perspective of information theory. In particular, the entropy of brain imaging time-series data has been well documented its technical advantages in the evaluation of several neurobiological disorders, including developmental dyslexia (Katan et al., 2017), depression (Zhao L. et al., 2019), epilepsy (Acharya et al., 2015), ADHD (Joy et al., 2021), and ASD (Milne et al., 2019). Therefore, as a main motivation, we aimed to test whether the entropy could be applied to measure eye movements during RAN.

Taken together, we suggested an entropy-based method to measure eye movements for the first time, which first mapped eye movements during RAN in a time-series and then analyzed the time-series by a proper definition of entropy from the perspective of information theory. By recruiting 408 children (206 males, aged 7–11 years), we aimed to: (i) Test whether the entropy of eye movements can gain better performance than traditional measures [e.g., total time of naming (TTN)] of eye movements; (ii) test whether the entropy of eye movements has a developmental trend in children across the age stages; (iii) test whether the entropy of eye movements may change with the task complexity of RAN; and (iv) investigate the association between the entropy of eye movements and the RAN-related skills evaluated by the number cancelation test (Xie et al., 2022), and between that and traditional measures (e.g., TTN) of eye movements.

## Materials and methods

All study procedures and research methods were carried out in accordance with the Declaration of Helsinki by the World Medical Association concerning human experimentation, and were approved by the Research Ethics Committee at Southeast University. Informed consent was obtained from all parents of participating children and oral consent was obtained from all participating children. Each child received an age-appropriate toy after completing the study.

### Study design and participants

The current study was conducted in Sanmenxia, Henan Province, China, between September 2021 to March 2022, and recruited 1,387 children (aged 7–11 years) from one primary school. Each child recruited was assigned a coding number according to the sequence number being selected randomly. Only children, whose coding number were with units digit 3, 6, or 9, were chosen to attend our experiments. Exclusion criteria were as follows: (a) Abnormal hearing functioning (i.e., hearing threshold levels bigger than 25 dB HL) and vision functioning (i.e., naked or corrected monocular visual acuities below than 1.0); (b) significant sensory or motor impairment; (c) a history of previous neurological or psychiatric disorders; (d) IQ score lower than 85 or bigger than 115; (e) children who had repeated a grade; and (f) incomplete measure data.

By steps above, a total of 408 children (206 males) attended the current experiments (see Table 1 for detailed information). Based on Weeks’s work (Weeks and Atlas, 2015), the sample size to be collected in the case of 95% confidence level and +5% accuracy was estimated to be *n* = 384, so this survey met the sampling requirement.

**TABLE 1:** Demographic characteristics of participants.

Age groups	Males (*N*, %)	Total (*N*)	Age (years)
7-years children	33 (50.00)	66	7.55 ± 0.24
8-years children	45 (47.87)	94	8.46 ± 0.28
9-years children	45 (54.88)	82	9.43 ± 0.28
10-years children	31 (45.59)	68	10.41 ± 0.30
11-years children	52 (53.06)	98	11.50 ± 0.28
Total	496 (50.49)	408	N/A

### Experimental tasks

#### Rapid automatized naming tasks

**FIGURE 1:**
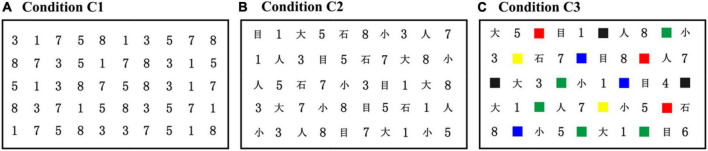
The C-RAN paradigm presented a 5 × 10 matrix of stimuli (e.g., numbers, Chinese characters, or colors) in different conditions: **(A)** condition C1 (i.e., naming a series of numbers sequentially); **(B)** condition C2 (i.e., naming a series of numbers and Chinese characters sequentially); and **(C)**. **(A)** Condition C1 (i.e., naming a series of numbers sequentially); **(B)** condition C2 (i.e., naming a series of numbers and Chinese characters sequentially); and **(C)** condition C3 (i.e., naming a series of numbers, Chinese characters, and colors sequentially). RAN, rapid automatized naming; C-RAN, Chinese RAN.

In the original RAN paradigm, a 5 × 10 matrix of stimuli (e.g., letters, numbers, colors, or objects) was visually presented, in which the matrix used five repetitions of each of the ten different stimuli with the order pseudo-randomized. Participants were required to name the stimuli sequentially as quickly and accurately as possible. To extend the application of RAN to developmental dyslexia in Chinese, we suggested a Chinese RAN (C-RAN) version, which substituted highly frequently used Chinese characters for English letters. As shown in Figure 1, the C-RAN paradigm in this study included three experimental conditions, depending on the format of stimuli to be named, i.e., Condition C1 (i.e., naming a series of numbers sequentially), Condition C2 (i.e., naming a series of numbers and Chinese characters sequentially), and Condition C3 (i.e., naming a series of numbers, Chinese characters, and colors sequentially).

Stimuli (shown in Figure 1) in each experimental condition were presented on a 21.5-in. TFT LCD monitor (1,920 × 1,080 resolution) with the participant seated approximately 60–90 cm away. A Tobii 4C (90 Hz; Tobii Technology AB, Danderyd, Sweden) eye tracker, calibrated using a standard 9-point grid, was used to measure eye movements. For each experimental condition, participants were required to name the stimuli (numbers, Chinese characters, or colors) as quickly and accurately as possible in a left-to-right and down fashion. To confirm that participants can understand the experimental rules and name the stimuli (numbers, Chinese characters, or colors) correctly, they were asked to do a preparatory task (i.e., a simplified C-RAN task with a 2 × 5 matrix of stimuli visually presented) before a formal experiment. Participants were excluded if their accuracy in the preparatory task was below than 0.8. It should be remarked that no participants were excluded in the current study due to their accuracy below than 0.8 in the preparatory task.

#### Number cancelation test

As a second experimental task, a number cancelation test (NCT) (Xie et al., 2022) was conducted to measure a participant’s RAN-related abilities. The examiner sat in front of a participant and presented the participant with a standard B5-size paper showing a series of numbers arranged in organized arrays with 26 rows and 40 columns. The participant, who hold a Digital Pen (with an embedded smart mini-camera), was required to find the number “3” (the targeted number) and then draw a circle on it, but ignore all other numbers (distractors), as quickly as possible within 2 min. The technical advantage of the Digital Pen was the usage of a smart mini-camera, designed to measure the temporal-spatial features from the perspective of handwriting kinematics, such as pre-movement time (initiating), movement time (moving pen to a stimulus), drawing time (completing a cancelation), circumference of a drawn curve, real-time spatial positions (trajectory) of drawing, and the time sequence of drawings (Xie et al., 2022). It should be remarked (Xie et al., 2022) that the temporal-spatial features may gain better performance than traditional measures of NCT (e.g., the number of omissions, the number of correct responses, the total number of cancelations, and completion time).

### Measures

#### Measures of number cancelation test

The Digital Pen (with an embedded smart mini-camera) can be used to measure a number of temporal-spatial features during NCT (Xie et al., 2022). In this study, we selected only three parameters (Xie et al., 2022) to evaluate individuals’ performance during the NCT. Those parameters were defined as follows.

(1) Speed of cognitive processing (SpC) was defined as:


(1) $$S\,p\,C=M\,\sum_{i=1}^{N}R_{i}$$

where *M* was the amount of numbers in one row (here *M* = 40); *N* was the total number of rows to be circled; *R*_*i*_1 represented the case if any number in the *i*-th row has been circled; and *R*_*i*_0 represented the case if no number in the *i*-th row had been circled.

(2) Selective Attention (SA) was defined as:


(2) $$S\,A=\frac{1}{T}\,\frac{m-\omega }{m+o}\times \text{SpC}$$

where *o* was the amount of omitted targets; ω was the number of distractors being circled; and *m* was the total amount of targets that should be circled; *T* was the task time (here *T* = 120); SpC was defined by Eq. (1).

(3) Averaged time of circlings (ATC) was defined as:


(3) $$A\,T\,C=\frac{1}{n}\times \sum_{i=1}^{n}t_{i}$$

where *n* was the amount of numbers being circled; and *t_i_* was the time to circle the *i*-th number.

#### Typical measures of eye movements

As verified in previous studies (Wiig et al., 2000), the mean percentage of naming accuracy remained stable and did not differ significantly for healthy children across the age stages. Hence, to evaluate individuals’ RAN abilities, the majority of studies and all published tests (Wolf et al., 2000; Georgiou et al., 2008; Norton and Wolf, 2012; Snowling and Melby-Lervag, 2016; Araújo and Faísca, 2019; McWeeny et al., 2022) considered only the TTN, which was widely used to measure the reading fluency and speed. Consequently, the current study considered the TTN, only, to evaluate eye movements during RAN.

#### Eye-movement entropy

Let (*x*_*t*_,*y*_*t*_) be the spatial coordinate of eye movements at the time *t*. Hence, *v_t_*, the velocity of eye movement at the time *t*, can be evaluated with the three-point central difference and thus be calculated by:


(4) $$v_{t}=\frac{\sqrt{{\left(x_{t+2}-x_{t+1}\right)}^{2}+{\left(y_{t+2}-y_{t+1}\right)}^{2}}-\sqrt{{\left(x_{t}-x_{t-1}\right)}^{2}+{\left(y_{t}-y_{t-1}\right)}^{2}}}{2\,\left(1/f_{s}\right)},$$

where *f_s_* is the sampling frequency.

For an experimental condition *C*_i_, the eye-movement entropy (EME) can be defined by:


(5) $$E\,M\,E\,\left(C_{i}\right)=-\sum_{j}p_{j}\,\left(v_{t}\right)\,\ln\,\left(p_{j}\,\left(v_{t}\right)\right),$$

where *v_t_* is defined in Equation (4); and *p*_*j*_(*v*_*t*_) is a probability distribution for *v*_*t*_0 corresponding to the case when a participant is naming a stimulus.

We generally required a data preprocessing before a formal calculation of EME. First, we roughly eliminated the contaminated data (e.g., extreme blink and saccade) according to the Teager Kaiser energy operator (Tran et al., 2014) and 3σ criterion. Then, we used a standard Hampel filter (Allen et al., 2010) (with window size of 6) to smooth the eye-tracking data. Finally, to convert the fixation into gaze, we adopted the Adaptive Piecewise Constant Approximation (APCA) algorithm (Chakrabarti et al., 2001) to slide and segment the eye-tracking data.

#### Entropy increase

The entropy increase *D*_ij_, which is caused by the change of experimental conditions from *C_j_* to *C_i_*, can be defined by:


(6) $$D_{i\,j}=E\,M\,E\,\left(C_{i}\right)-E\,M\,E\,\left(C_{j}\right).$$

#### Influence of small perturbation of eye movements on eye-movement entropy measures

To reveal the performance difference between EME and traditional measures (e.g., TTN) of eye movements, we randomly added “small perturbation” (e.g., rapid movements between focuses in the presence of skipping or omitting some stimulus during RAN) with different amplitude to the velocity of the original eye movement, under the condition that TTN remained unchanged. Then, we aimed to test whether EME may reflect the amplitude change of “small perturbation” of eye movements.

### Statistical analysis

**FIGURE 2:**
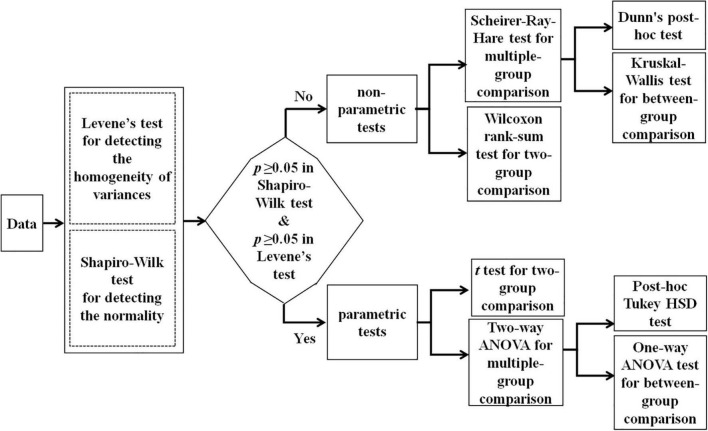
The flowchart of statistical analysis.

As the first target, we aimed to investigate how the age and gender influence EME during C-RAN tasks. For this purpose, we conducted a series of two-way ANOVA for EME in different experimental conditions, according to the flowchart shown in Figure 2. As shown below, we verified that our data (i.e., EME measures) failed to pass both normality test and variance homogeneity test, so we carried out a series of non-parametric two-way ANOVA procedures (i.e., Scheirer-Ray-Hare tests). In addition, we applied the Kruskal Wallis method and Dunn’s *post-hoc* test for multiple comparisons with Benjamini-Hochberg procedure to control the false discovery rate.

As noted, C-RAN task in this study actually included three experimental conditions. As the second target, we expected to understand how the experimental conditions and gender influence the EME increase. For this purpose, we conducted a series of two-way ANOVA, according to the flowchart shown in Figure 2. Again, we found that our data failed to pass both normality test and variance homogeneity test, so we carried out a series of non-parametric tests and used multiple comparisons with controlled false discovery rate.

As the third target, we used the Pearson’s approach to investigate the association between EME and measures of the NCT (Xie et al., 2022), and that between EME and traditional measure (e.g., TTN) of eye movements in three conditions.

Finally, we expected to test whether EME may be more sensitive to small perturbation of eye movements than traditional eye-movement measures (e.g., TTN).

All statistical analysis above was conducted with R language (version 4.0.2).

## Results

### General information of participants

The current study investigated a total of 408 children, including 206 males and 202 females. The ratio of males to females was 1.01:1 and the participants were distributed in 5 age groups, see Table 1 for detailed information. We verified that there was no significant gender difference (χ^2^ = 1.81, *p* = 0.77).

### Main effects of age and gender on eye-movement entropy

As a main motivation, we aimed to investigate how the age and gender influence EME. According to the statistical flowchart shown in Figure 2, we verified that our data (i.e., EME measures) failed to pass both normality test and variance homogeneity test (*p*’s ≥ 0.05). Hence, we conducted a series of non-parametric two-way ANOVA procedures (i.e., Scheirer-Ray-Hare tests) to reveal the age and gender main effects as well as for their interaction. Our findings showed that in all experimental conditions, the main effect of age was significant, but the main effect of gender was not significant, and there was no any interaction effects (Gender: *p*’s > 0.05; Age: *p*’s < 1 × 10^–4^; Gender*Age: *p*’s > 0.05).

**FIGURE 3:**
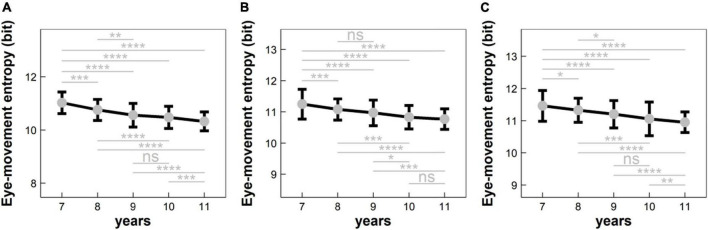
Eye-movement entropy changed with the age in different conditions: **(A)** condition C1 (i.e., naming a series of numbers sequentially); **(B)** condition C2 (i.e., naming a series of numbers and Chinese characters sequentially); and **(C)** condition C3 (i.e., naming a series of numbers, Chinese characters, and colors sequentially). ns: *p* > 0.05; *p < 0.05; **p < 0.01; ***p < 1 × 10^– 3^; ****p < 1 × 10^–4^.

According to the statistical flowchart shown in Figure 2, we further utilized the Kruskal Wallis method and Dunn’s *post hoc* for multiple comparisons with Benjamini-Hochberg procedure to control the false discovery rate. Figure 3 summarized our results and verified that:

(1) For EME in Condition C1 (see Figure 3A): (i) EME significantly deceased with the age in a monotonic progression (*p*’s < 0.05, adjusted); (ii) EME did not differ significantly between 9- and 10-years children (*p* > 0.05), implying that there was a developmental plateau for 9–10-years children.

(2) For EME in Condition C2 (see Figure 3B): (i) EME significantly deceased with the age in a monotonic progression (*p*’s < 0.05, adjusted); (ii) EME did not differ significantly between 8- and 9-years children and between 10- and 11-years children (*p*’s > 0.05), implying that there was a developmental plateau for both 8–9- and 10–11-years children.

(3) For EME in Condition C3 (see Figure 3C): (i) EME significantly deceased with the age in a monotonic progression (*p*’s < 0.05, adjusted); (ii) EME did not differ significantly between 9- and 10-years children (*p* > 0.05), implying that there was a developmental plateau for 9–10-years children.

### Entropy changed with task complexity

C-RAN task in this study included three experimental conditions, i.e., Conditions C1, C2 and C3. It is clear from Figure 1 that the task complexity will be increased if the experimental condition is changed from C1 to C2, from C1 to C3, or from C2 to C3. We aimed to investigate how the experimental conditions and age influenced the entropy increases *D*_*ij*_ defined in Eq. (6).

According to the statistical flowchart shown in Figure 2, we again verified that our data (the values *D*_*ij*_) failed to pass both normality test and variance homogeneity test (*p*’s ≥ 0.05). Hence, we conducted a series of non-parametric two-way ANOVA procedures to reveal the condition and gender main effects as well as for their interaction. Our findings showed that: (i) The main effect of condition was significant in all age stages, but the main effect of gender was not significant in all age stages (Gender: *p*’s > 0.05; Condition: *p*’s < 1 × 10^–4^); and (ii) there was a significant interaction effect between gender and condition in 7-years children (*p* < 0.05), but there were not in other ages (*p*’s > 0.05).

**FIGURE 4:**
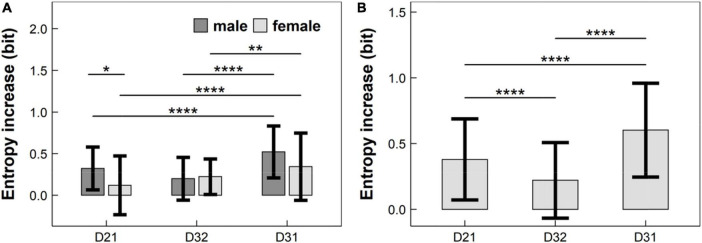
Entropy increases caused by the change of experimental conditions in: **(A)** children aged 7 years; and **(B)** children aged 8–11 years, where entropy increases *D*_21_, *D*_32_, and *D*_31_, defined in Equation (6), were corresponding to the EME change from Condition C1 to C2, from Condition C2 to C3, and from Condition C1 to C3, respectively. EME: eye-movement entropy; *: *p* < 0.05; **: *p* < 0.01; ****: *p* < 1 × 10^–4^.

According to the statistical flowchart shown in Figure 2, we utilized non-parametric multiple comparisons with controlled false discovery rate. Figure 4 summarized our results and verified that:

(1) For 7-years children (see Figure 4A): The entropy increase *D*_21_ in males was significantly lower than that in females (*p* < 0.05, adjusted), but the entropy increases *D*_32_ and *D*_31_ did not differ significantly between males and females (*p’s* > 0.05, adjusted);

(2) For 7-years males (see Figure 4A): There was a significant difference between *D*_21_ and *D*_31_ (*p* < 1 × 10^–4^, adjusted), and between *D*_32_ and *D*_31_ (*p* < 1 × 10^–4^, adjusted);

(3) For 7-years females (see Figure 4A): There was a significant difference between *D*_21_ and *D*_31_ (*p* < 1 × 10^–4^, adjusted), and between *D*_32_ and *D*_31_ (*p* < 0.01, adjusted);

(4) For children aged 8–11 years (see Figure 4B): There were significant differences among *D*_21_, *D*_32_ and *D*_31_ (*p’s* < 1 × 10^–4^, adjusted).

### Correlation analysis

To illustrate the effectiveness of EME, it is crucial to analyze the correlation between EME and other abilities related with RAN. Table 2 summarized our results and showed that: (i) There was a significant negative association between SA and EME in three experimental conditions with *r* between –0.43 and –0.36 (*p’s* < 1 × 10^–4^); (ii) There was a significant negative association between SpC and EME in three experimental conditions with *r* between –0.38 and –0.30 (*p’s* < 1 × 10^–4^); and (iii) There was a significant positive association between ATC and EME in three experimental conditions with *r* between 0.28 and 0.36 (*p’s* < 1 × 10^–4^).

**TABLE 2:** Correlations between measures of the NCT and EME in three conditions.

	Condition C1	Condition C2	Condition C3
SA	–0.43****	–0.36****	–0.36****
SpC	–0.38****	–0.30****	–0.30****
ATC	0.36****	0.28****	0.28****

****p < 10^–4^.

EME, eye movement entropy; NCT, number cancelation test.

Furthermore, by Pearson’s correlation method, we analyzed the association between both EME and TTN in different experimental conditions during C-RAN. Table 3 summarized our results and showed that there was a significant positive association between EME and TTN in all experimental conditions with *r* between 0.95 and 0.97 (*p*’s < 1 × 10^–4^).

**TABLE 3:** Correlations between EME and TTN in three experimental conditions.

	Correlation coefficient
Condition C1 (i.e., naming a series of numbers sequentially)	0.97****
Condition C2 (i.e., naming a series of numbers and Chinese characters sequentially)	0.97****
Condition C3 (i.e., naming a series of numbers, Chinese characters, and colors sequentially)	0.95****

****p < 10^–4^.

EME, eye-movement entropy; TTN, total time of naming.

### Advantages of eye-movement entropy

**FIGURE 5:**
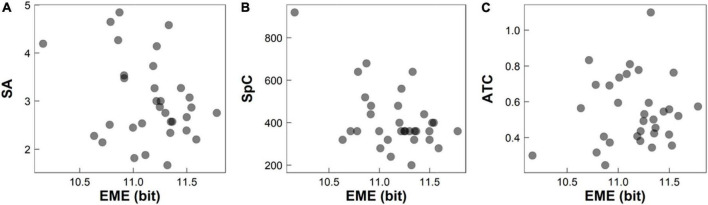
Participants had the same TTN (e.g., TTN = 18 s) during RAN but had different EME, SA, SpC, and ATC: **(A)** EME vs. SA; **(B)** EME vs. SpC; and **(C)** EME vs. ATC. EME, eye-movement entropy; SA, selective attention; SpC, speed of cognitive processing; ATC, averaged time of circlings. TTN, total time of naming; RAN, rapid automatized naming.

TTN measures the total time to complete the whole RAN task, and is proven to be strongly related with the reading fluency and speed. However, TTN ignores many dynamical details during RAN, and cannot monitor the focus points in sequence and record the essential visual activities during RAN, such as pauses over informative regions of interest, rapid movements between focuses, scanning path, the presence of skipping or omitting, and temporal-spatial series of focuses. Hence, even when two children have the same TTN, they both might have different reading-related abilities. In the meanwhile, EME has been well-documented to reflect the complexity and irregularity of a system and illustrated its technical advantages in the evaluation of several neurobiological disorders. Therefore, it is not surprising that EME can obtain better performance than TTN in evaluating individual’s RAN abilities. Figure 5 illustrated a case that participants had the same TTN (e.g., TTN = 18 s) but had different reading-related abilities, such as EME, selective attention (measured by SA), cognitive speed (measured by SpC), and visual-motor integration skill (measured by ATC). This indicates that EME might be more sensitive to evaluate reading-related abilities than TTN (ignoring many dynamical details).

**FIGURE 6:**
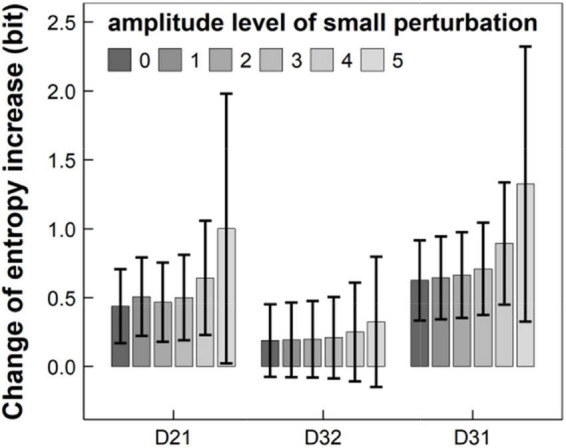
Entropy increases *D*_21_, *D*_32_, and *D*_31_ changed with the amplitude of small perturbation, ranging from level 0 to 5, where TTN remained unchanged. TTN, total time of naming.

Furthermore, we randomly added “small perturbation” (e.g., rapid movements between focuses in the presence of skipping or omitting some stimulus during RAN) with different amplitude to the velocity of the original eye movement, under the condition that TTN remained unchanged. Figure 6 summarized our results in 11-years children and showed that the entropy increases *D*_*ij*_ basically increased with the amplitude of small perturbation, but TTN remained unchanged. This implies that the entropy may reflect small perturbation of eye movements and thus have higher sensitivity, but traditional eye-movement measure (e.g., TTN) may not.

## Discussion

Eye tracking methodology (Jones et al., 2008; Armstrong and Olatunji, 2012; Association, 2013; Chawarska et al., 2013; Lai et al., 2013; Hogan-Brown et al., 2014) has the feasibility to characterize the essential features of RAN. However, traditional measures of eye movements ignore many details about the visual and cognitive processing of RAN, and are usually associated with the duration of time spent on some particular areas of interest, first fixation, fixation counts, revisited fixation counts, saccadic velocities, or saccadic amplitudes. To cope with the drawback of traditional measures, we suggested an entropy-based method to measure eye movements for the first time, which first mapped eye movements during RAN into a time-series (containing detailed dynamical information of eye movements), and then analyzed the time-series by a proper definition of entropy. Findings showed that the entropy of eye movements was more sensitive to reflect small perturbation (e.g., rapid movements between focuses in the presence of skipping or omitting some stimulus) of eye movements during RAN, and thus gained better performance than traditional measures (e.g., TTN). This may be interpreted by the fact that the entropy, reflecting the complexity and irregularity of a system, has well-documented its clinical utility in the evaluation of several neurobiological disorders. We also confirmed that the entropy of eye movements significantly deceased with the age and the task complexity of RAN.

To illustrate the effectiveness of EME measure, we required to test whether there were strong correlations between EME and traditional measures of eye movements, as well as that between EME and other RAN-related skills. Findings showed that: (i) There was a significant positive association between EME and TTN in all experimental conditions (*p*’s < 1 × 10^–4^); (ii) There were significant negative associations between SA and EME, and between SpC and EME in three conditions (*p’s* < 1 × 10^–4^); and (iii) There was a significant positive association between ATC and EME in three conditions (*p’s* < 1 × 10^–4^). This implies that children with lower EME might have lower TTN and ATC, and higher SA and SpC. On the other hand, individuals with lower TTN may have higher RAN abilities; while, individuals with lower ATC and higher SA and SpC may have better performance during NCT. Hence, children with lower EME may have higher RAN abilities and better performance during NCT; while, children with higher EME may have lower RAN abilities and worse performance during NCT. This finding supports that the EME increase or reduction might be considered as a feature to identify the difference of RAN between typically developmental children and children with developmental dyslexia or learning disabilities.

The NCT was suggested to measure some RAN-related abilities, which involved cognitive skills in selective and sustained attention, motor inhibition, visuospatial search, planning, organizing, psychomotor speed, intact visual-perception abilities, fine motor coordination, and sensory motor integration (Xie et al., 2022). It seems in theory that both RAN and NCT may share several visual and cognitive neural circuits because they both need a similar “visual scanning” processing. In addition, NCT and RAN are associated with “writing” and “reading,” respectively. Hence, it is hypothesized that RAN, in combination with NCT, may bring some new insights into the understanding of developmental dyslexia and learning disabilities (Benjamin et al., 2019).

RAN has been well studied in the evaluation of several cognitive and neurobiological disorders, including developmental dyslexia (Goswami, 2015), language disorders (Snowling and Melby-Lervag, 2016), ADHD (Tannock et al., 2000), and ASD (Hogan-Brown et al., 2014; Zhao J. et al., 2019). In particular, previous meta-analyses have verified the significant correlation between RAN and reading across various reading constructs and languages (Swanson et al., 2003; Araújo et al., 2015; Hjetland et al., 2017), and thus, RAN might predict future reading across different ages, ability levels, and languages. More importantly, RAN deficits might become even more prominent in interpreting and characterizing the features of developmental dyslexia than other deficits in cognitive skills, such as phonological awareness, short-term memory, letter knowledge, and vocabulary (Wolf et al., 2000; Georgiou et al., 2008; Norton and Wolf, 2012; Snowling and Melby-Lervag, 2016; Araújo and Faísca, 2019; McWeeny et al., 2022). Therefore, it is significant to reveal the essential mechanisms underlying RAN. As noted, our research was carried out along this technical direction, and confirmed that the entropy of eye movements may provide more perspectives and deeper understanding of RAN.

Due to potential applications (Swanson et al., 2003; Araújo et al., 2015; Hjetland et al., 2017) of RAN to interpret cognitive and neurobiological disorders, it is crucial to gain the normative data of RAN across a wide range of ages. However, this question has not been well investigated (Wiig et al., 2000; Hjetland et al., 2017). Indeed, thus far, there is an American normative data (Wiig et al., 2000), only. Even though the current study did not focus on a Chinese normative data of RAN, it still provided sufficient referenced information about the Chinese normative data in children aged 7–11 years, as well as how the age and gender influenced the Chinese normative data. In particular, our findings showed in children aged 7–11 years that: (i) EME during RAN deceased significantly with the age in a monotonic progression, implying a trend of entropy reduction with the increase of age; and (ii) EME during RAN did not differ significantly between males and females for all age stages. This is the first time to report such a result associated with the Chinese normative data of RAN. Remarkably, we showed that there were developmental plateaus in abilities in Condition C1 (i.e., naming a series of numbers sequentially) and Condition C3 (i.e., naming a series of numbers, Chinese characters, and colors sequentially) for 9–10-years children, and in abilities in Condition C2 (i.e., naming a series of numbers and Chinese characters sequentially) for 8–9- and 10–11-years children. In addition, our finding is consistent with previous studies (Wolf et al., 2000; Georgiou et al., 2008; Norton and Wolf, 2012; Snowling and Melby-Lervag, 2016; Araújo and Faísca, 2019; McWeeny et al., 2022) that main effect of gender is not significant in RAN tasks. It is clear that: (i) The entropy increase *D*_21_, corresponding to the EME change from Condition C1 (i.e., naming a series of numbers sequentially) to Condition C2 (i.e., naming a series of numbers and Chinese characters sequentially), represents the influence of Chinese characters on the C-RAN; (ii) The entropy increase *D*_32_, corresponding to the EME change from Condition C2 (i.e., naming a series of numbers and Chinese characters sequentially) to Condition C3 (i.e., naming a series of numbers, Chinese characters, and colors sequentially), represents the influence of colors on the C-RAN; and (iii) The entropy increase *D*_31_, corresponding to the EME change from Condition C1 (i.e., naming a series of numbers sequentially) to Condition C3 (i.e., naming a series of numbers, Chinese characters, and colors sequentially), represents the influence of Chinese characters and colors on the C-RAN. Findings (see Figure 4) showed that *D*_31_ was lower than *D*_21_ and *D*_32_; and *D*_21_ was lower than *D*_32_. This implies that EME might increase with the task complexity of RAN. This inference is consistent with the previous researches (Åvall et al., 2019; Georgiou and Parrila, 2020) that alphanumeric RAN (e.g., naming numbers or letters) may have higher cognitive complexity and activate a wider range of brain regions than non-alphanumeric RAN (e.g., naming colors or objects). It should be remarked (Åvall et al., 2019; Georgiou and Parrila, 2020) that alphanumeric RAN tasks are more strongly related to future reading than non-alphanumeric tasks.

It is natural to adopt a Chinese version of RAN in the understanding of developmental dyslexia in Chinese. The difference between both the original RAN and Chinese version is due to the features of Chinese characters: (i) Chinese characters not only have shape and sound attributes like English letters, but also represent meaning; (ii) Chinese characters have no clear form-to-sound conversion rules, so readers need to remember the pronunciation of Chinese characters; and (iii) The visual complexity of Chinese characters are much higher than that of English letters. Consequently, compared with the original RAN, the Chinese version may have higher cognitive complexity, and thus activate a wider range of brain regions (Liao et al., 2015; Peng et al., 2017). To extend the application of RAN to developmental dyslexia in Chinese, we suggested a Chinese version of RAN (i.e., the C-RAN) by substituting Chinese characters (highly frequently used) for English letters. We expect that the C-RAN should be more suitable in the evaluation of developmental dyslexia in Chinese than the original RAN.

The entropy can be used to reflect the complexity and irregularity of a system from the perspective of information theory. Its advantages have been well documented in the analysis of brain imaging time-series for the evaluation of neurobiological disorders, including developmental dyslexia (Katan et al., 2017), depression (Zhao L. et al., 2019), epilepsy (Acharya et al., 2015), ADHD (Joy et al., 2021), and ASD (Milne et al., 2019). In particular, it has been verified (Acharya et al., 2015; Katan et al., 2017; Milne et al., 2019; Zhao L. et al., 2019; Joy et al., 2021) that the higher the entropy, the higher the complexity and irregularity of the brain will be. This inference has been widely applied to better understand the entropy abnormality in different context. Remarkably, we showed for the first time that the entropy may be extended to measure eye movements during RAN, and gain better performance than traditional measures.

The entropy reduction is a well-known and well-acceptable principle in physics, and thus can be easily used for data interpretation. For instance, we can infer from the entropy reduction principle that: (i) The RAN abilities may increase with the age, then the entropy of eye movements during RAN may decrease with the age; (ii) Individuals with higher RAN-related abilities may generally have lower entropy of eye movements during RAN; and (iii) RAN tasks with higher complexity may generally activate bigger entropy of eye movements. As noted above, those inferences have been verified in the current study. We expect that the entropy reduction principle can be applied to reveal the abnormities of RAN deficits and developmental dyslexia. Because eye tracking technique is fundamental for psychological research, the entropy-based measure method suggested in this study may have the potential to be used in a very broad prospect of applications.

## Conclusion

This article suggested an entropy-based method to measure eye movements for the first time, which first mapped eye movements during RAN in a time-series and then analyzed the time-series by a proper definition of entropy from the perspective of information theory. Findings showed that EME gained better performance (e.g., more sensitive to reflect “small perturbation” of eye movements during RAN) than traditional measures, and decreased with the age and the task complexity of RAN. In addition, this study also verified that EME significantly correlated with traditional eye-movement measure (e.g., TTN) and the RAN-related skills (e.g., SA, cognitive speed, and visual-motor integration). Our findings may bring some new insights into the understanding of both RAN and eye tracking technique itself.

## Data availability statement

The raw data supporting the conclusions of this article will be made available by the authors, without undue reservation.

## Ethics statement

The studies involving human participants were reviewed and approved by the Research Ethics Committee at Southeast University. Written informed consent to participate in this study was provided by the participants or their legal guardian/next of kin.

## Author contributions

DY and HW developed the idea for the study. HW, FL, and YD collected the data. HW and DY did the analyses. DY wrote the manuscript. All authors contributed to the article and approved the submitted version.
